# The Role of Pathogenic *E. coli* in Fresh Vegetables: Behavior, Contamination Factors, and Preventive Measures

**DOI:** 10.1155/2019/2894328

**Published:** 2019-11-26

**Authors:** J. J. Luna-Guevara, M. M. P. Arenas-Hernandez, C. Martínez de la Peña, Juan L. Silva, M. L. Luna-Guevara

**Affiliations:** ^1^Engineering in Food, Faculty of Chemical Engineering, Benemerita Universidad Autonoma de Puebla, 14 South and San Claudio Avenue, University City, San Manuel, CP 72590, Puebla, Mexico; ^2^Center for Research in Microbiological Sciences, Institute of Sciences, Benemerita Universidad Autonoma de Puebla, 14 South and San Claudio Avenue, University City, San Manuel, CP 72590, Puebla, Mexico; ^3^Food Safety & Processing, Mississippi State University, Starkville, MS 39762, USA

## Abstract

Many raw vegetables, such as tomato, chili, onion, lettuce, arugula, spinach, and cilantro, are incorporated into fresh dishes including ready-to-eat salads and sauces. The consumption of these foods confers a high nutritional value to the human diet. However, the number of foodborne outbreaks associated with fresh produce has been increasing, with *Escherichia coli* being the most common pathogen associated with them. In humans, pathogenic *E. coli* strains cause diarrhea, hemorrhagic colitis, hemolytic uremic syndrome, and other indications. Vegetables can be contaminated with *E. coli* at any point from pre- to postharvest. This bacterium is able to survive in many environmental conditions due to a variety of mechanisms, such as adhesion to surfaces and internalization in fresh products, thereby limiting the usefulness of conventional processing and chemical sanitizing methods used by the food industry. The aim of this review is to provide a general description of the behavior and importance of pathogenic *E. coli* in ready-to-eat vegetable dishes. This information can contribute to the development of effective control measures for enhancing food safety.

## 1. Introduction

The consumption of fresh produce has increased notably in recent years due to multiple contributions of nutrients and functional properties [1, 2]. Over the last 30 years, there has been a 25% increase in the average amount of fresh produce consumed per person in the USA [3]. A diet rich in fruit and vegetables has been shown to protect against various types of cancer and chronic illnesses, such as coronary heart disease [4]. However, at the same time, consumption of fresh produce is associated with a growing number of foodborne outbreaks due to bacterial contamination of these products [5].

Leafy greens, such as lettuce, spinach, and fresh herbs, are some of the vegetables most frequently linked to bacterial infections [6]. In the United States, from 1990 to 2005, the Food Safety Project reported that at least 713 produce-related outbreaks were associated with foodborne disease, of which 12% involved fresh fruits and vegetables [4, 7]. In 2011, the Advisory Committee on the Microbiological Safety of Food (ACMSF) reported that, in the UK, there were 531 cases of reported illness, including one death, related to the consumption of fruits and vegetables between 2008 and 2010 [8]. In the same year, Germany reported an outbreak of Shiga toxin-producing *E. coli* (STEC) serotype O104 : H4; at the end of the outbreak, 3785 cases of illness were reported outside of Germany, identifying contaminated sprouted seeds as responsible for the foodborne outbreak [4].

It should be emphasized that the effect of foodborne diseases affects not only the sick person but also has considerable economic repercussions. On the one hand, there are costs related to the sick person, including medical care and absenteeism from work and school. On the other hand, there are the costs on society, including the decrease in worker productivity, expenses of research on the outbreak, the loss of income due to food companies closing, legal expenses for litigation related to diseases, and the expenses in public medical services [9].

It has been shown that how crops are harvested, processed, and distributed has enhanced both the supply and variety of products, which may also have increased the risk of more widespread outbreaks. The increase in illness associated with consumption of fresh produce reflects a documented increase in food contamination [10].

Foodborne illness may be the cause of fresh produce contamination by pathogenic bacteria, viruses, and protozoa [11–14]. This contamination may originate from manure, soil, sewage, surface water, or wildlife [15]; it may also occur during washing, slicing, soaking, packing, and food preparation [3]. Among the bacteria associated with foodborne illnesses are *Listeria monocytogenes* [16], *E. coli* [17], *Shigella soney* [18], *Salmonella* [19], and *Staphylococcus aureus* [20].

Survival and growth of these microorganisms depend on several factors, including the specific features of the microorganism, fruit ripeness, environmental conditions, plant development, bacterial resistance to the plant metabolic processes, plus harvest, and postharvest processes [21]. Particularly, some pathogenic microorganisms can internalize and adhere to the plant surface [15]. Unfortunately, current industrial sanitizing and washing treatments of fruits and vegetables (e.g., triple washing of prepackaged leafy greens) do not guarantee the total elimination of pathogens [22]. Therefore, this review considers the main *E. coli* pathotypes associated with foodborne outbreaks due to fresh produce consumption.

Furthermore, some recently introduced processes, considered to prevent the contamination of raw vegetables, are also described. They range from the production stages to the hygienic conditions during food preparation, from “the field to the table.” Reij and Den Aantrekker [23] reported that important factors contributing to the presence of pathogens in prepared foods are insufficient hygiene (1.6%), cross contamination (3.6%), processing or storage in inadequate rooms (4.2%), contaminated equipment (5.7%), and contamination by personnel (9.2%).

## 2. Incidence of *E. coli*

The most common vegetables associated with *E. coli* STEC are sprouts and green leafy vegetables (Table 1). The possible source of the contamination of sprouts is the seed that is used (it was possible to see that there were many contaminated seed lots). In the case of leafy greens, it appears that contaminated water (drag water from cattle lots or water contaminated by other sources) is the most common source of contamination. Many outbreaks reported around 30 cases, with the ratio of hospitalizations to cases ranging from 18 to 67%.

## 3. Contamination Factors in Fresh Vegetables

There are three types of factors that affect microbiota present in fresh products: physical, chemical, and biological. Physical factors, such as pH, temperature, and moisture, affect the growth and some metabolic activities of microbiota. Chemical factors include the availability and nutrients in vegetables that may be used by microorganisms. Finally, biological factors include the presence of competitive microbiota and bacterial-plant interactions [24]. Fresh produce may be contaminated at any point in the production chain between farm and table. It has been shown that produce contamination is high during three periods: in the field, during initial processing, and in the kitchen [25].  Table 2 lists agricultural factors (organic fertilizer, irrigation water, soil, and spraying of pesticides and insecticides) and postharvest practices (handling, collection, washing, processing, transportation, and packaging) that can cause the contamination of raw vegetables by various pathogenic microorganisms, including *E. coli* [12, 26–28].

### 3.1. Preharvest Factors

Soil and improperly composted animal manure are considered to be the main preharvest contamination factors. Soil is a natural reservoir for a large variety of human pathogens, including pathogenic *E. coli*, due to the addition of animal waste [29]. *E. coli* O157 : H7 may survive in the soil from 7 to 25 weeks depending on soil types, humidity level, and temperature. This bacterium can also survive during crop storage or distribution [30]. According to Launders et al. [31], the presence of STEC O157 in potatoes represents a risk because it may cause cross contamination with other foods that are consumed raw. Furthermore, in organic food production, the use of animal manure is a common practice; several reports relate this type of crop system to the presence of fecal contamination, particularly during the leafy vegetable harvest [32]. According to the Centers for Disease Control and Prevention (CDC) [33], several US states were affected by the consumption of organic spinach contaminated with STEC O157.

Domestic animals and wildlife also represent a potential source of pathogenic bacteria, particularly for lettuce and leafy greens at preharvest stages along the coast of California and in Yuma, AZ [34]. Berger et al. [10] showed that the feces of wildlife are involved in vegetable contamination and may cause *E. coli* O157 : H7 outbreaks. Jay-Russell et al. [6] studied a potential reservoir for pathogenic *E. coli* in feces from coyotes and dogs. Insects could also be a source of plant contamination. Contaminated flies have been shown to transfer *E. coli* to plant leaves or fruits [10]. In addition, Lynch et al. [18] found that intensive agricultural practices have forced crop fields to be too close to animal production areas. The ecological consequences of this proximity have increased the likelihood of contamination by *E. coli* O157 : H7 in wildlife: the percentages tested positive in unspecified duck was 5% (1/20 total samples) in Washington, USA; in large mammals including deer, such as the black-tailed deer (*Odocoileus hemionus columbianus*), it was 11.1% (1/9 total samples); in California, USA, in unspecified deer, it was 25% (1/4 total samples); in Ireland, in feral pig (*Sus scrofa*), it was 14.9% (13/87 total samples); in California and in small mammals in England, such as the rabbit (*Oryctolagus cuniculus*), it was 48.8% (20/41 total samples). All sample types were feces, anal and cloacal swabs, or gastrointestinal contents from individual animals, unless otherwise noted [35].

Seasons are another important environmental condition that affects the prevalence of *E. coli* in vegetables. For example, *E. coli* contamination in cilantro and parsley significantly increased in fall compared to that found in spring and winter [18]. The finding of *E. coli* in irrigation water has been associated with the presence of feces from cattle and other animals, especially during heavy rainfall.

There are current reports on outbreaks caused by the consumption of lettuce irrigated with water contaminated with *E. coli* O157 : H7 [36]. However, the risk associated with the use of contaminated water for irrigation depends on the irrigation system used. There is a lower probability risk of spreading pathogens from contaminated water through drip irrigation versus overhead sprinkler systems [37]. Another study shows that well water used for irrigation may be contaminated with *E. coli* O157 : H7 from feces of cattle or other animals, which can be observed especially during heavy rainfall. Also, karst formations occur when acidic water begins to break down bedrock surfaces, allowing surface water to enter fractures in limestone, contaminating the groundwater, which then favors the survival of *E. coli* in karst streams for long periods [17].

An additional factor during the handling and harvesting of crops are the workers' hands. They can become a vehicle for contamination during preharvest due to the lack of access to latrines or handwashing stations [18].

### 3.2. Postharvest Contamination

In some cases, the presence of *E. coli* in vegetables, such as alfalfa sprouts, fresh spinach, and raw clover sprouts, is significantly higher at final postharvest stages compared to early stages of handling [38]. This may be due to subsequent direct contamination or by pathogen multiplication during postharvest procedures in raw vegetables.

The confirmation of *E. coli* in postharvest packing steps indicates possible fecal contamination and the potential presence of enteric pathogens of fecal origin. According to Zhang et al. [39], when *E. coli* O157 : H7 was isolated from certain types of fresh vegetables, the prevalence was relatively low, but this microorganism can cause illness in consumers.

Water is employed in many steps, such as washing, chill tanks, sprays, and shipping ice during the postharvest process. The washing procedure is required to remove soil and debris from vegetables and some microorganisms. In spite of this, if the water used is contaminated, washing, slicing, soaking, packaging, and preparation may be the original source of *E. coli* transmission to vegetables. The use of contaminated water in hydrocoolers in which fresh products are stored may generate vegetable contamination [40]. Other sources of potential contamination with *E. coli* during the preparation of green leafy vegetables (salads) include the water baths or dump tanks used by packers and the lack of cooling during storage [30]. In addition, food contamination may occur if the vegetables are prepared with unclean implements in restaurants or home kitchens. Lynch et al. [18] mentioned that the establishment of pathogens, such as *E. coli*, in vegetables may occur through cross contamination by the food handler's hands due to poor hygiene when raw meat or poultry are also being prepared.

Some outbreaks have been associated with the cutting of vegetables during salad preparation. The fresh-cut produce used in the salads has been linked to the bacterial growth. Estrada-Garcia et al. [41] discussed the risk of acquiring an ETEC infection when chopped vegetables are used in the preparation and consumption of Mexican salsa. Due to the great diversity of possible sources of contamination in fresh vegetables, more studies are required to learn how to prevent and correct contamination during pre- and postharvest processing.

### 3.3. Preharvest and Postharvest Preventive Measures for Fresh Produce

During preharvest, some pathogens may be transferred to the environment by application of inadequately composted animal manure [10]. Therefore, it is essential to use fertilizers that are properly “stabilized.” One way to stabilize them is through the use of composting, in which the organic matter is decomposed by the action of microorganisms for a certain period of time (e.g., 3 or 15 days) at a designated temperature (131°F), followed by a stage of curing under colder conditions. These conditions reduce the levels of pathogenic microorganisms, promote the decomposition of cellulose and lignin, and stabilize their composition [42, 43]. Untreated human sewage should not be used to fertilize vegetables and crops for human consumption [27], unless it complies with the specifications for the use of biosolids according to regulation [44].

There is a risk of microbial contamination from water associated with irrigation systems due to the relationship between the volume of water retained on the crop's surface, the amount of food consumed, and time harvest [45, 46]. Likewise, there is a recognized need to establish GAPs (Good Agricultural Practices) based on produce safety standard protocols for the irrigation of fresh produce [47]. During postharvest, wash water can be a transmission vehicle for pathogens, especially when this water is reused [28]. In addition, *E. coli* can survive for relatively long times in tap water, which can have serious consequences for the health of consumers. This point was revealed in incidents occurring in the water supply system of Walkerton, Canada, which was contaminated with *E. coli* O157 : H7; seven people died, and more than 2,300 people became ill [48].

In addition, the risk of reclaimed water may be reduced through treatment and disinfection systems, such as activated charcoal, reverse osmosis, membrane filtration, chlorination, ozonation, and UV irradiation; however, some systems are often expensive, particularly in developing countries [45].

Postharvest treatment of fruit and vegetables is also involved in food contamination; these treatments include handling, storage, transportation, and cleaning. Various studies reveal that food workers were frequently engaged in unsafe food handling, promoting microbial contamination of ready-to-eat foods. This typically occurs because food handlers are asymptomatic carriers of pathogenic microorganisms or have poor personal hygiene. Measures to diminish the risk of contamination by food workers include implementing proper handwashing and improving personal hygiene [20].

The World Health Organization [49] suggests 5 basic steps to prevent contamination of food by *E. coli* and other enteropathogens: (1) separating raw and cooked foods, (2) keeping the work area clean, (3) cook (the food thoroughly), (4) keeping food at safe temperatures, and (5) using safe water and raw materials.

Other actions to decrease food contamination are the use of better disinfectants. Recently, studies examined different and novel disinfectants for produce disinfection, such as chlorine dioxide, ozonized water, and electrolyzed oxidizing water. However, all these methods have their own limitations, making them unsuitable for an extensive application [50]. For example, ozonized water has been approved as GRAS (generally recognized as safe) by the FDA (Food and Drug Administration) as an effective disinfectant against bacteria, fungi, protozoa, and microbial spores; however, ozone is very unstable and may be toxic, causing eye and respiratory system irritation [51]. Other alternatives are those proposed by Qi et al. [50], using sodium persulfate activated by ferrous sulfate and sodium hydroxide, which effectively inactivate *E. coli* O157 : H7. According to the Food Safety Modernization Act (FMSA) for fresh products, food handlers should receive education on the appropriate use of sanitizing agents and on the principles of food hygiene and safety [52]. Another important measure is for managers to be well trained in microbiology, so they can properly supervise preparation of the agents. These training measures could contribute to the reduction of foodborne disease outbreaks associated with the consumption of raw vegetables.

While the most commonly used sanitizer is chlorine at 100 to 200 ppm, other alternative sanitizers, including ozone, peroxyacetic acid, and chlorine dioxide, are actively being evaluated for efficacy against pathogenic and spoilage microorganisms. Peracetic acid (80 ppm), chlorine (100 and 200 ppm), chlorine dioxide (3 and 5 ppm), and ozone (3 ppm) reduce populations >5 log of *E. coli* O157 : H7 inoculated on apples, lettuce, strawberries, and cantaloupe. Sensory panels only detected the use of 80 ppm peracetic acid on chopped lettuce and 200 ppm sodium hypochlorite on whole apples, with the other treatments being acceptable for consumers [53]. Angeles-Núñez [54] showed that poor hygiene in containers and transportation may be sources of contamination and may be counteracted with an adequate system of washing, disinfection, and the application of good agricultural practices. The main risk factors of contamination during transportation include following improper production practices, temperature abuse, unsanitary cargo areas, improper loading or unloading procedures, damaged packaging, shipping containers in inadequate condition, poor employee habits, and road conditions [55].

Another approach, the use of Modified Atmosphere Packaging (MAP) of fresh fruits and vegetables results in an extended shelf life. MAP systems generally utilize an internal package atmosphere other than air in a hermetically sealed package of suitable permeability. O_2_, CO_2_, and N_2_ are the most commonly employed. The effect of MAP in inhibiting the growth of pathogens is more influenced by the type of vegetables than by the particular gas used. According to the study conducted by Abadias et al. [22], the population of *E. coli* O157 : H7 was higher in fresh-cut carrots (7.0–8.4 log cfu·g^−1^) at 25°C after 3 days of storage, while in fresh-cut melon, the bacterium reached populations of 8.5 and 8.9 log cfu·g^−1^ after 1 day of storage; in modified atmosphere packaging, no growth was observed in the fresh-cut pineapple.

As mentioned in the previous section, the food processing industry has been using chemical decontamination (hypochlorite, peroxyacetic acid, organic acid, hydrogen peroxide, trisodium phosphate, and ozone) and physical decontamination (gamma irradiation) of ready-to-eat fresh produce. However, it has been recently reported that the nonthermal method of pulsed ultraviolet (PUV) light is a more effective method for reducing EHEC biofilm on fresh produce and packaging materials. A different strategy is focused on the use of plant commensal microbiota to compete with pathogens for diffusible factors or carbon sources in vegetal leaves and roots [4, 10, 56–59].

Recent studies are focusing on improving the efficacy of antimicrobial agents by increasing the lethal activity on pathogenic microorganisms such as *E. coli*, specifically focusing on the toxicity of reactive oxygen species (ROS) such as superoxide, hydrogen peroxide, and hydroxyl radical. These agents usually accumulate after exposing the bacteria to a stressor agent, such as an antimicrobial. According to Hong et al. [60], the blocking of ROS accumulation by exogenous mitigating agents slowed or inhibited the *E. coli* poststressor death, and they concluded that the lethal action of the agents depends in part of an amplifying accumulation of ROS that exceeds primary damage repair.

## 4. Survival Conditions and Persistence Mechanisms

*Escherichia coli* is an innocuous member of the human and warm-blooded animal gut microbiota; however, pathogenic strains may cause intestinal and extraintestinal infections. These primary hosts may acquire *E. coli* from water and food contaminated with feces; therefore, the presence of *E. coli* is used as an indicator of fecal contamination.

Some *E. coli* strains have been isolated from various plants used for human consumption, and these plants, such as spinach, lettuce, alfalfa, cress, bean, arugula, tomato, and radish, are considered a secondary host [61–64]. These plants have physical barriers such as wax, cuticle, cell wall, trichomes, and stomata (natural pores). It has been shown that some bacteria use stomata as entrance points to the leaf interior. Several human pathogenic bacteria can survive on and penetrate the plant interior in the apoplast; they can remain in this environment with low metabolic activity, and they are able to survive drastic changes in temperature, pH, osmolality, and nutrient deprivation [65, 66].

Pathogenic *E. coli* possess adherence factors for human epithelial colonization, and it has been shown that several of these factors are also used for adherence to raw vegetables [67]. On the contrary, the plant offers *E. coli* a harsh environment with aerobic conditions, lower temperature, low pH, a high level of UV (ultraviolet) energy, and aerial surfaces (phyllosphere), which are poor in nutrients and contain antimicrobial secondary metabolites [68]. However, diarrheagenic *E. coli* have evolved mechanisms for vegetal attachment that vary according to the strain and plant involved (Table 3). Contamination of raw vegetables with *E. coli* is important since vegetables are used for fresh food preparations and since low doses of infection are sufficient to cause intestinal disease (*E. coli* O157 : H7 <100 or even <10) [64].

### 4.1. STEC *E. coli* O157 : H7

Verotoxigenic or Shiga-like toxigenic *E. coli* (VTEC or STEC) O157 : H7 is considered a large threat in foodborne diseases. *E. coli* O157 : H7 became the first of several strains referred to as enterohaemorrhagic *E. coli* or EHEC, which can produce one or more Shiga toxin (also called verocytotoxins and formerly known as Shiga-like toxins). STEC strains can survive in fresh ground beef and on fresh leafy green vegetables, and it is well known that the main reservoirs for VTEC are ruminants, which continually shed bacteria into the environment, contaminating food and water.

Various mechanisms involving adhesins, fimbriae, flagella, and the LEE-encoded effectors are typically used by VTEC for colonization and attachment [80]. LEE is a chromosomal region called the locus of enterocyte effacement that encodes a type III secretion factor (T3SS), an adhesion called intimin (*eae*), and the translocated receptor of intimin [81]. However, little is known about the use of these mechanisms in how VTEC is interacting with the plants as secondary hosts.

Several studies have shown that several proteins or structures are needed to colonize different plants. For example, it was reported that VTEC requires the EspA fiber of T3SS, but not pili or flagella to attach to arugula leaves [69] or the T3SS to colonize lettuce and spinach [70]. However, it needs the T3SS, flagella, curli, *E. coli* common pilus (ECP), and the hemorrhagic coli pilus (HCP) to colonize baby spinach leaves [71]. Curli is also implicated in the adherence of EHEC to alfalfa sprouts. This amyloid fimbria is also involved in binding to, and invasion of, human epithelial cells [72, 73].

There are several genes and/or structures associated with biofilm formation for plant colonization by STEC. These biofilms, which are formed of poly-beta-1,5-*n*-acetyl-D-glucosaminecellulose, cellulose, and colonic acid, are found in sprouts and tomato root segments [74]. In order to determine what type of genes are differentially expressed during the plant colonization, Crozier et al. [82] performed a transcriptomic analysis following exposure of VTEC O157 : H7 to plant extracts, specifically those of spinach (*S. olercera*) or lettuce (*Lactuca sativa*). Interestingly, genes differentially regulated are associated with metabolism, biofilm, stress response, and some unknown genes. On the contrary, genes for the pathogenicity island LEE and motility, which have a role in the pathogenicity in humans, are repressed.

*E. coli* O157 : H7 has the ability to colonize and internalize mainly in live spinach and lettuce plants [10, 83–86]. It can be retained (bound or entrapped) by several parts of the plant, such as leaves, sprouts, and fruit, even after vigorous washing or disinfection [61, 87–90]. For example, EHEC has the ability to adhere diffusely to the epidermis, with aggregation around the stomata, and penetration to a depth of 20 to 100 *μ*m into the stomata and junction zones (spongy mesophyll) of cut lettuce leaves [10, 91]. In addition, it has been shown that *E. coli* O157 : H7 can move into the plant through the root system to reach the edible portion of lettuce [89]. In accordance with the European Food Safety Authority and the World Health Organization [49, 92], the only way to eliminate STEC from food is through heating (cooking to 70°C). On the contrary, Lu et al. [57] reported that bacteria of the *E. coli* group were reduced to less than 30 MPN/g, which was the safety level, when fresh-cut celery were irradiated with 1.0 kGy.

### 4.2. Non-O157 : H7 STEC

Although there are 200 different serotypes of STEC, few are associated with foodborne illness. In Mexico, some non-O157 STEC strains were identified in raw foods, such as vegetable salads and carrot juice [3, 93]. Some clinically relevant non-O157 : H7 serotypes are commonly called “the big six”; they include serotypes O26, O45, O103, O111, O121, and O145 [94]. In a recent study with non-O157 Shiga toxin-producing *E. coli* (STEC) strains that included the big six (O26, O45, O103, O111, O121, and O145), three other serogroups implicated in serious illness (O91, O113, and O128) from humans, sheep, or bovines showed the ability to form biofilms on abiotic surfaces. These strains also exhibited resistance to multiple antimicrobials [95]. This biofilm forming capability could be important for STEC persistence in the environment, and it could also contribute to the presence of reservoirs for antibiotic resistance genes.

### 4.3. ETEC

Enterotoxigenic *E. coli*, ETEC, is an important cause of infantile and travelers' diarrhea [80]. ETEC's virulence factors are the heat labile toxin (LT) and the heat stable toxin (ST). Both mediate deregulation of ion channels in the epithelial cell membrane [96]. ETEC can survive in a variety of environments, such as rivers, drinking water, irrigation water, and fresh vegetables [97]. It has been shown that ETEC has the ability to form biofilms through curli and extracellular matrix (1,5-*n*-acetyl-D-glucosaminecellulose, cellulose, and colonic acid) on sprouts and tomato roots [74, 75]. Also, ETEC adheres firmly to lettuce and leafy vegetables through flagella [76]. It has been observed that the growth of enteric pathogens such as ETEC is greater in plant tissue having mechanical damage due to availability of nutrients. Finally, ETEC in contact with various parts of the plant or its extracts can differentially regulate the expression of its genes; for example, this bacterium responds depending on its exposure to lysates or to acidic pH in vegetables [98].

### 4.4. EAEC

Enteroaggregative *E. coli* (EAEC) is the second most common cause of travelers' diarrhea after ETEC and is associated with foodborne outbreaks of diarrhea in developing countries. The main virulence factors are the heat stable toxin (EAST1), the Shigella enterotoxin (ShET1), and hemolysin E [41]. There is a Shiga toxin-producing EAEC strain responsible for one of the largest foodborne outbreaks in Europe, the EAEC strain O104 : H4. This strain combines the chromosomal backbone of EAEC with a bacteriophage encoding Stx2 from STEC [99].

Recently, Borgersen et al. [100] found that pathogenic Shiga toxin EAEC O104 : H4 produces 3- to 6-fold higher levels of exopolysaccharide structures of colonic acid than the EAEC O42 strain. This colonic acid structure and the biofilm structures are formed on the surface of sprouts. Expression of the colonic acid capsule, in O42 and O104 : H4, is seen at room temperature, but not at 37°C. In addition, bile downregulates the expression of colonic acid. All of this suggests that EAEC exists in a “biofilm competent state” in the environment, which promotes its persistence on raw vegetables. When EAEC enters the human gastrointestinal tract, the bacterium is protected from the acid pH of the stomach by the biofilm that covers it [100]. Later, biofilm expression is downregulated by bile at 37°C, and other important virulence factors are expressed at the intestinal level. Production by colonic acid is not exclusively a property of EAEC: EHEC, EPEC, and *E. coli* enteroinvasive (EIEC) are also able to produce colonic acid.

EAEC also shows adherence to the arugula leaf (*Eruca vesicaria*) as two different phenotypes, one of small bacterial aggregates covering the entire leaf surface and the other of dense bacterial attachment to the guard cell that surrounds the stomata. Using mutant analysis, both phenotypes are explained through the aggregative adherence fimbriae (AAF) and the flagella. The flagellum mutant adheres to the epidermis but lacks stomatal tropism. In contrast, an *aaf* mutant lacks the ability to adhere to the epidermis while maintaining stomatal adherence. In this way, multiple adherence factors are involved in the interaction of EAEC to raw vegetables [63].

## 5. Conclusion

The presence of enteropathogenic bacteria in fresh produce plays an important role in the emergence of foodborne outbreaks. There are many possible sources of contamination on fresh produce due to exposure to many different environments and handling. More studies are necessary to better understand how to prevent the occurrence of *E. coli* on fresh produce. The attachment to plant surfaces is the first step in the colonization process and subsequent transmission of pathogens via the edible parts of plants. However, each enteropathogen has its own molecular mechanisms of adherence and fitness to the vegetable biosphere; many are similar to mechanisms used to colonize the primary host. All enteropathogens survive in fresh produce for commercially relevant periods despite the use of multiple disinfection systems. The future of food safety lies in adherence to strategies for the different categories of *E. coli* pathogens. These measures will help prevent bacterial transmission and benefit human health. Finally, growers, producers, packers, and food consumers need to examine their own processes and incorporate strategies for maintaining food safety.

## Figures and Tables

**Table 1:** Selected *E. coli* 0157 : H7 (otherwise noted) outbreaks associated with vegetables reported by the CDC.

Year	Vehicle	Cases	Hospitalizations	Deaths	Description and possible source(s)
2018	Romaine lettuce	210	96	5	Whole genome sequencing implicated *E. coli* 0157 : H7 found in canal water from the Yuma, AZ region. Could have been the result of water contaminated by water from cattle feedlot found upstream
2017	Leafy greens	25	9	1	Associated with various leafy green types
2016	Alfalfa sprouts	11	2	0	Associated with one sprout facility. The source is not known, but the contaminated seed could have been the source
2014	Raw clover sprouts—*E. coli* O121	19	8	0	FDA concluded that the source could have been a lot of contaminated seed sanitary deficiencies found in the installation
2013	Ready-to-eat salads	33	7	0	Salads contained cooked chicken/ham which could have been the source of contamination
2012	Mixed organic spinach and spring salad	33	13	0	It was traced back to a facility, but the source of contamination is unknown
	Raw clover sprouts—*E. coli* O26	29	7	0	Was traced to a possible lot of contaminated seeds
2011	Romaine lettuce	49	33	0	Traceback led to a farm that no longer produced the lettuce, and thus, the source is unknown
2010	Shredded romaine lettuce—*E. coli* O145	30	12	0	Traceback could not lead to the source of contamination
2006	Fresh washed spinach	199	102	3	Similar samples of *E. coli* were isolated from water and cattle manure from a nearby cattle ranch The presence of wild pigs on the ranch and the proximity of surface waterways to wells could have also played a role

Available at http://www.cdc.gov/ecoli/outbreaks.html.

**Table 2:** Contamination sources during the pre- and postharvest stages in raw vegetables.

Stage	Factor
Preharvest	Soil, irrigation water, inadequately composted manure, human handling, reconstituted fungicides and insecticides, seasons (fall, winter, and spring)
Postharvest	Harvesting equipment, transport container, contaminated water used for washing, transport vehicles and processing equipment, unclean implements, poor hygiene in hands, cross contamination (preparation or storage)

**Table 3:** *Escherichia coli* strains associated with foodborne diseases and the factors involved for plant colonization.

Patotype	Serotype	Food outbreak year	Vehicle	Virulence factors involved in attachment to raw vegetables
EHEC	Sakai	1996	White radish sprout	T3SS (EspA) arugula T3SS (lettuce and spinach) T3SS, curli, flagellum, *Enterohemorrhagic E. coli* common pilus (baby spinach leaves) Curli (alfalfa sprout) Biofilm sprouts and tomato root
	O157 : H7	1998	Lettuce	
		1999	Lettuce	
	O157 : H7	2006	Spinach Iceberg lettuce	
	
		2008 X2	Lettuce	
		2008	Spinach	
		2009	Spinach	
		2011	Romaine lettuce	
		2012	Spring mix and spinach	
		2013	Ready-to-eat salad	
EHEC	O26	2012	Raw clover sprouts	
	O121	2014	Raw clover sprouts	
	O104 : H4		Fenugreek sprouts	Colonic acid capsule
ETEC		2008	Sprouts and tomato roots	Biofilm of 1,5-*n*-acetyl-D-glucosaminecellulose, cellulose, colonic acid, and curli
		2011	Lettuce and leafy vegetables	Flagella
EAEC				Flagellar adhesion and Afa I/II

References: [63, 69–79].
